# Cardiovascular diseases in Brazil: premature mortality, risk factors and priorities for action. Comments on the preliminary results from the Brazilian National Health Survey (PNS), 2013: Doenças cardiovasculares no Brasil: mortalidade prematura, fatores de risco e prioridades de ação. Comentários sobre os resultados preliminares da Pesquisa Nacional de Saúde (PNS), 2013

**DOI:** 10.1590/1516-3180.2015.13320018

**Published:** 2014-11-28

**Authors:** Paulo Andrade Lotufo

**Affiliations:** I MD, DrPH. Titular Professor, Discipline of Internal Medicine, Faculdade de Medicina da Universidade de São Paulo (FMUSP), São Paulo, Brazil.

## SITUATION #1: UNDERSTANDING CARDIOVASCULAR EPIDEMIOLOGY IN BRAZIL

1. Mortality: Cardiovascular diseases - coronary heart disease (CHD) and stroke - are the main cause of death worldwide.^1^ Over recent decades, Brazil has witnessed a decline in mortality rates due to CHD and stroke.^2^^,^^3^ However, in 2012, these diseases were the first and the third commonest causes of premature death nationwide, respectively.^1^ Table 1 ^4^^,^^5^^,^^6^ shows the proportion of deaths among individuals under the age of 70 years due to all cardiovascular diseases, CHD alone and the combination of stroke and hypertensive disorders, according to sex and race, in Brazil in 2012. The proportion of premature deaths due to CHD is higher than the proportion due to stroke for both sexes and all races. Men die due to all cardiovascular events earlier than women. According to skin color/race, the chance of death under the age of 70 years is highest for black individuals, followed by mixed race and white people. To understand this picture better, so as to enable effective intervention, more information concerning morbidity and risk factors is necessary.
2. Incidence and case-fatality: The morbidity due to cardiovascular diseases can be ascertained by determining the incidence and case-fatality rates. Population-based incidence rates have been obtained from studies limited to a single city.^7^ Case-fatality rates are easier to obtain from the organized hospital-based registries that have been created in Brazil.^8^^,^^9^^,^^10^ These registries are helpful in determining the levels of pre-hospital care, compliance with guidelines among the medical team and, especially, quality of procedures such as thrombolytic therapy, angioplasty and coronary revascularization. In addition, data on the adherence among patients who suffered cardiovascular events, regarding the advice that they were given about diet, exercise and use of medicines, can be obtained. In summary, hospital-based registries are important for improving the quality of medical care, but they do not help to create preventive programs. Strategies for preventive action depend on obtaining a certain minimum knowledge regarding the prevalence of morbidity and risk factors at the population level.
3. Prevalence: Determining the prevalence of cardiovascular risk factors is not an easy task in a country that is as large as Brazil. However, during 2013, the Ministry of Health carried out the first national representative survey addressing health conditions, morbidity and risk factors among a sample of Brazilians over the age of 18 years, called “Pesquisa Nacional de Saúde” (PNS), or the Brazilian National Health Survey. The preliminary results were released in December 2014, including new information about hypertension, dyslipidemia, diabetes and smoking habits.^11^ Table 2 summarizes the main results, which will be detailed below.

**Table 1.**
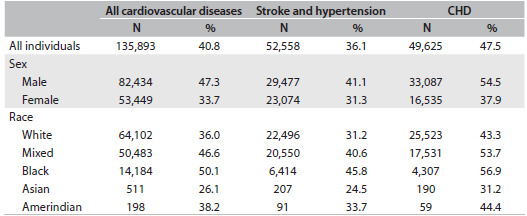
Numbers and proportions of deaths among individuals under the age of 70 years due to all cardiovascular diseases, stroke and hypertension and coronary heart disease (CHD), in comparison with the events that occurred at all ages

**Table 2.**
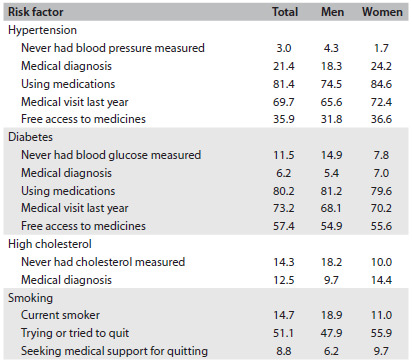
Description of risk factors evaluated in the first Brazilian National Health Survey, 2013

## SITUATION #2: CARDIOVASCULAR RISK FACTORS IN THE NATIONAL HEALTH SURVEY

1. Hypertension: This is the most significant risk factor for morbidity and mortality due to stroke in Brazil, despite the reduction in the risk of death. The proportion of individuals who have never had a blood pressure measurement made is insignificant. The prevalence of medical diagnoses among individuals over the age of 18 years is approximately the same as observed in earlier localized surveys in Brazil. However, the most important finding is that among people who self-declare as “hypertensive”, 80% are taking medications. It was found that one-third of the people with high blood pressure had free access to antihypertensive drugs. Men and women did not have the same behavior regarding medical visits and use of medicines.
2. Diabetes: Plasma or blood glucose measurements were made at least once for approximately 90% of the participants. A medical diagnosis of diabetes was made in 6.2%. If it is accepted that half of the people with diabetes do not know about this situation until they are tested,^12^ the presumptive prevalence of 12% thus obtained is relatively close to that of other recent surveys in Brazil.^13^^,^^14^ Likewise, 80% of the individuals with hypertension were underusing medicines. In contrast, the proportion of diabetics receiving drugs free of charge was significantly different from the proportion of individuals with hypertension.
3. Dyslipidemia: The participants were asked about cholesterol and/or triglyceride measurements, and 85% confirmed that they had been tested for dyslipidemia during their lifetimes. A medical diagnosis of dyslipidemia had been made in the cases of 14.3% of the participants, with a significant difference between the sexes.
4. Smoking: The survey identified an astonishing prevalence of current smoking: 18.9% among men and 11% among women. Almost half of them were trying to quit, but fewer than 10% had sought medical advice and support.

## COMMENT #1: THE NATIONAL HEALTH SYSTEM IS ALSO WORKING TOWARDS A “HEALTHY HEART”

The general view concerning cardiovascular risk factors is optimistic. The National Health System that was created in 1988 has implemented primary care and family health programs and this, together with better risk management within the private sector, is providing greater access for diagnosing and treating cardiovascular risk factors. This is having an impact on cardiovascular disease rates.^15^^,^^16^ Free-of-charge access to medicines for hypertension and diabetes, which was launched by the National Health System in 2007, is showing good results despite controversy regarding the portfolio of antihypertensive medicines.^17^

Another interesting point is that the “delayed cardiovascular epidemiological transition in Brazil”, i.e. the preponderance of mortality due to stroke over mortality due to CHD, has vanished.^18^ On the other hand, the racial gap regarding cardiovascular mortality is not exclusive to hypertension and stroke.^19^ As shown in Table 1, deaths due to CHD are more premature among blacks than among whites.

## COMMENT #2: THERE HAS BEEN AN IMPRESSIVE PUBLIC-HEALTH VICTORY IN RELATION TO CURBING THE SMOKING EPIDEMIC

The most import finding is the lower prevalence of current smokers compared with three decades ago.^20^ This has certainly had an impact on the reduction of cardiovascular and respiratory mortality in Brazil. One important point to consider in this regard is that the effects of laws to restrict and advertising to restrain the smoking habit are probably at the limit of their efficacy. The reason is that most of these 15% who continue to smoke are in fact addicted to nicotine. This implies that there is a need for organized actions within primary care settings, such as psychological support and free availability of effective drugs for supporting nicotine withdrawal.^21^^,^^22^

## COMMENT #3: DYSLIPIDEMIA NEEDS TO BE THE PRIORITY FOR PRIMARY CARE PROVIDERS

The priorities of the program for controlling hypertension and diabetes were established 15 years ago. However, as indicated in Table 1, the proportion of premature CHD in Brazil is higher than that of stroke. Dyslipidemia has been recognized as the most important risk factor for CHD since the time of the first results from the Framingham Heart Study.^23^ In addition, the impact of lipid-lowering agents has been very well documented in randomized clinical trials relating to primary and secondary prevention.^24^ The recent and controversial guidelines from the American Heart Association/American College of Cardiology recommend that statins should be prescribed. Moreover, they recommend that instead of considering cholesterol levels in isolation, the overall cardiovascular risk should be assessed by adding information about blood pressure, diabetes and smoking to the cholesterol data.^25^ In contrast to two decades ago, when statins first came onto the market, statins today are generic drugs with relatively low cost and they need to be included in the free-of-charge access program for cardiovascular prevention.

In conclusion, the scientific community, public and private healthcare administrators and the entire National Health System need to combine their efforts to combat the burden of heart diseases. The first Brazilian National Health Survey will be a very useful tool for cardiology prevention in this country.
